# A novel transcriptional regulator, CdeR, modulates the type III secretion system via c-di-GMP signaling in *Dickeya dadantii*

**DOI:** 10.1128/spectrum.02655-24

**Published:** 2025-03-05

**Authors:** Alaleh Ghasemi, Xiaochen Yuan, Ching-Hong Yang

**Affiliations:** 1 Department of Biological Sciences, University of Wisconsin-Milwaukee, Milwaukee, Wisconsin, USA; 2 Department of Plant Pathology, Entomology and Microbiology, Iowa State University, Ames, Iowa, USA; Pennsylvania State University, University Park, Pennsylvania, USA

**Keywords:** Helix-Turn-Helix, bacterial second messenger, soft rot, diguanylate cyclase, potato

## Abstract

*Dickeya dadantii* is a bacterial pathogen that causes soft rot disease in many plant species worldwide, including temperate, subtropical, and tropical regions. This bacterium employs the type III secretion system (T3SS) to manipulate host immune responses. Although cyclic-di-GMP (c-di-GMP), a ubiquitous bacterial second messenger, negatively regulates the expression of T3SS genes in *D. dadantii*, the underlying mechanism remains unclear. In this study, we identified a potential transcriptional regulator, CdeR, which regulates the T3SS involving c-di-GMP. Through transposon mutagenesis, we discovered that deletion of *cdeR* in a *gcpD* mutant background restored T3SS gene expression. GcpD is a diguanylate cyclase responsible for c-di-GMP synthesis, and its deletion led to high T3SS gene expression due to low c-di-GMP. Further analysis revealed that, in the *gcpD* mutant background, CdeR regulates T3SS by manipulating intracellular c-di-GMP levels, involving another diguanylate cyclase, GcpL, whose expression is upregulated by CdeR. Additionally, we found that removing helical regions within the Helix-Turn-Helix DNA-binding domain of CdeR completely disrupted its regulation of the T3SS, underscoring the essential role of this domain in CdeR’s functional activity. This study is the first to identify CdeR as a potential transcriptional regulator involved in T3SS regulation. Our findings provide significant insights into the regulatory mechanisms of T3SS and highlight the complex interactions between bacterial second messengers and transcriptional regulators in pathogenic bacteria.

**IMPORTANCE:**

Bacterial pathogens, such as *Dickeya dadantii*, must adapt to diverse environmental and host conditions by utilizing intricate regulatory networks to control virulence. This study identifies CdeR, a novel transcriptional regulator, as a crucial factor in modulating the expression of the type III secretion system (T3SS), a key virulence mechanism. Importantly, we show that CdeR operates in a cyclic-di-GMP (c-di-GMP)-dependent manner, linking this second messenger to T3SS regulation in *D. dadantii* for the first time. Our findings reveal a sophisticated interaction between c-di-GMP signaling and transcriptional regulation, highlighting how these systems collectively drive bacterial virulence. This work advances our understanding of bacterial pathogenesis and opens new avenues for developing targeted strategies to mitigate soft rot disease in crops, potentially improving agricultural productivity and plant health.

## INTRODUCTION

The bacterial plant pathogen *Dickeya dadantii* causes soft rot, wilt, and blight diseases in a wide range of important crop species, including potato, tomato, cabbage, and chicory (1, 2). In general, *D. dadantii* can be found on plant surfaces, soil, as well as water‐logged environments, such as streams and rivers (3, 4). This widespread distribution poses a significant challenge for disease management. The pathogenicity of *D. dadantii* relies heavily on its ability to produce cell-wall-degrading enzymes, including cellulases, pectinases, proteases, and polygalacturonases (5, 6). The type III secretion system (T3SS), a conserved protein translocation system that delivers bacterial effector proteins into host cells, is a crucial virulence factor for *D. dadantii*. This system effectively modulates the host defense response, allowing for efficient colonization by the bacteria (7, 8).

The regulation of the *D. dadantii* T3SS involves at least two distinct two-component systems (TCSs): HrpX/HrpY and GacA/GacS. At the transcriptional level, HrpX/HrpY activates T3SS expression by upregulating the transcription of *hrpL*, which encodes the T3SS master regulator (9). *hrpL* transcription is activated by the binding of RpoN (a sigma factor σ^54^) to its promoter region, with stimulation from the enhancer-binding protein HrpS (10). Subsequently, the alternative sigma factor HrpL binds to the *hrp* box region (GGAACC-N_15/16_-CCACNNA) and initiates the transcription of downstream T3SS genes like *hrpA*, *hrpN*, and *dspE*, encoding the T3SS needle protein, harpin, and virulence effector, respectively. The *hrpL* transcript is also post-transcriptionally regulated by the RsmA/*rsmB* system. RsmA, a small RNA-binding protein, binds the 5′ untranslated region of *hrpL* mRNA and promotes its degradation (11). The TCS GacA/GacS activates the transcription of *rsmB*, a non-coding RNA containing several RsmA binding sites, which binds RsmA to neutralize its negative effect on *hrpL* mRNA (12).

The bacterial second messenger, bis-(3′−5′)-cyclic dimeric guanosine monophosphate (c-di-GMP), acts as a global signaling molecule, regulating T3SS expression through several c-di-GMP associated components in *D. dadantii* (13–17). Two different types of enzymes control intracellular c-di-GMP levels: diguanylate cyclase (GGDEF domain-containing DGCs) and phosphodiesterase (EAL or HD-GYP domain-containing PDEs) are responsible for synthesizing and breaking down c-di-GMP, respectively (18, 19). c-di-GMP serves as a versatile ligand that binds to different effectors, including PilZ domain proteins, degenerate GGDEF or EAL domain proteins, transcription factors, and RNA riboswitches to regulate specific cellular responses (20). Consequently, the dynamic intracellular concentration of c-di-GMP enables bacterial cells to adapt to changing environments and initiate responses (18, 21–25).

Genomic analysis of *D. dadantii* revealed 12 GGDEF-domain-encoding genes, 4 EAL-domain-encoding genes, and 2 EAL-GGDEF-dual-domain-encoding genes (15). Deletion mutants targeting each GGDEF and/or EAL domain protein-encoding gene exhibited various phenotypes, suggesting specific roles for individual DGCs/PDEs in regulating different cellular behaviors or virulence. For example, GcpA, a DGC, was found to negatively regulate pectate lyase production via the H-NS protein and *rsmB* regulatory RNA, while it negatively regulated T3SS expression through a different pathway, bypassing the H-NS and Rsm system (15).

Transcriptional factors are categorized based on the structural characteristics of their DNA-binding domains (26). The most abundant DNA-binding domain in prokaryotes is the Helix-turn-Helix (HTH) domain (27). So far, several families of transcription factors have been identified in *D. dadantii*, and numerous promoters have been determined to be under their control. For example, HrpY binds to the *hrpS* promoter region, positively regulating its transcription (28). The SlyA protein, a MarR family transcriptional regulator, binds to the promoter region of the *phoP* gene, which encodes the sensor kinase in the PhoP-PhoQ TCS (29). The LysR-type transcriptional regulator PecT suppresses the expression of pectate lyase genes at high temperatures by directly binding to their promoters (30). In this study, we first revealed the biological function of GcpD in *D. dadantii*, showing that GcpD negatively regulates the expression of T3SS genes. Further, we identified a putative transcriptional regulator, ABF-0020041, that plays a critical role in GcpD-mediated T3SS regulation. In a *gcpD* mutant background, ABF-0020041 represses the expression of *gcpL*, another gene encoding a DGC, resulting in elevated expression of T3SS encoding genes. Consequently, we renamed ABF-0020041 as CdeR, based on its role as a c-di-GMP-dependent regulator.

## RESULTS

### GcpD is a negative regulator of the T3SS gene expression in *D. dadantii*

**Fig 1:**
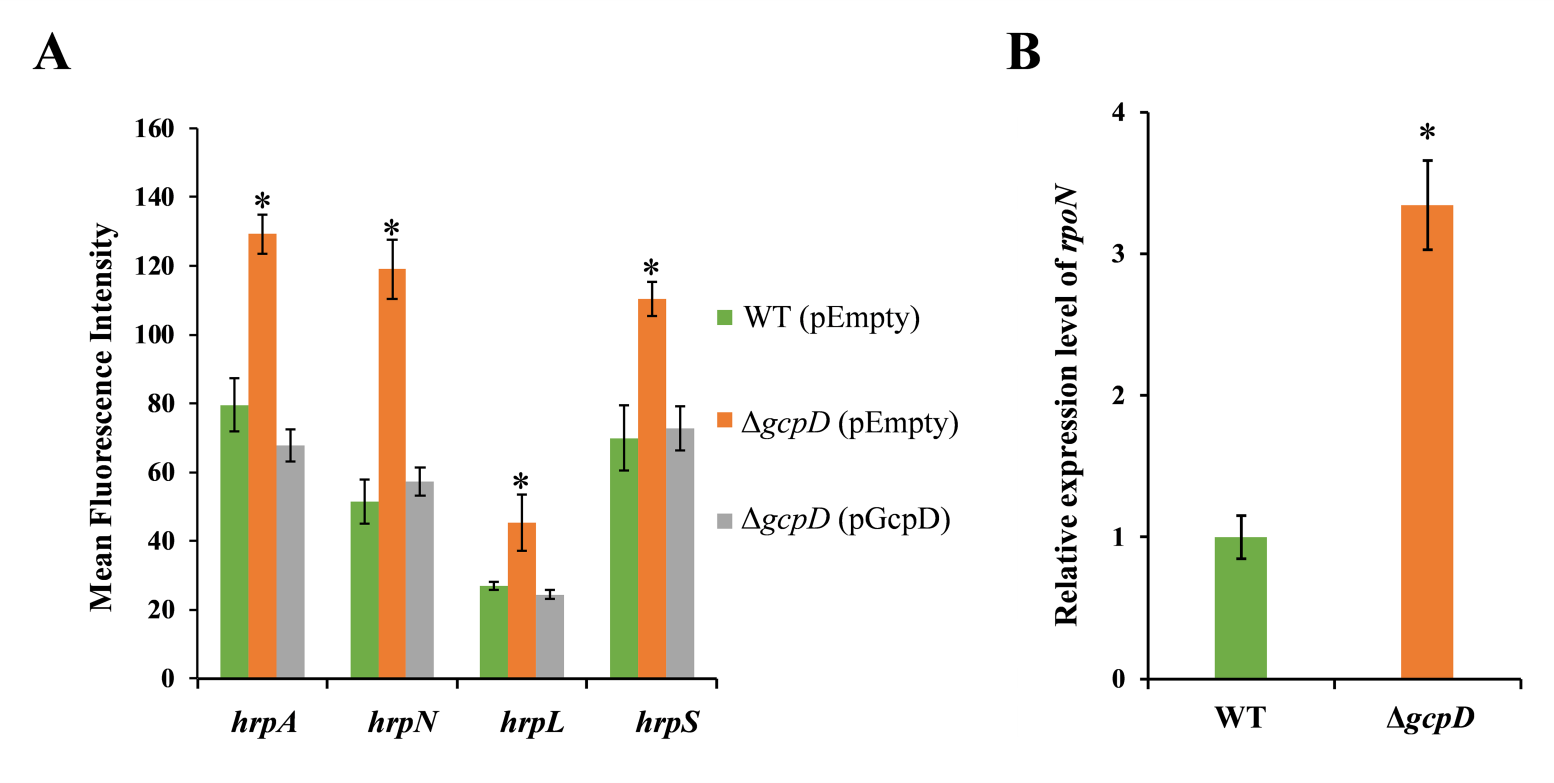
GcpD controls the expression of T3SS genes. (**A**) The promoter activities of *hrpA*, *hrpN, hrpL*, and *hrpS* were measured in wild-type (WT) *D. dadantii* harboring empty vector pCL1920, Δ*gcpD* harboring pCL1920, and Δ*gcpD* harboring pCL1920-*gcpD*, respectively. (**B**) The RNA levels of *rpoN* were examined in WT bacteria and Δ*gcpD*. Experiments were repeated three times with similar results, and each experiment had triplicates. Error bars indicate standard errors of the means. Asterisks indicate statistically significant differences in the means (*P* < 0.05 by Student’s *t* test).

In our previous study, we demonstrated that deletion of gene *gcpA*, encoding a DGC, led to increased *hrpA* expression relative to wild-type (WT) *D. dadantii* (15). GcpD, a putative DGC, shares similar N-terminal sensory domains with GcpA, including GAF (cGMP phosphodiesterase, Adenyl cyclase, and FhlA domain) and PAS (Per/Arnt/Sim), the cellular function of which remains unknown. To investigate whether GcpD affects the T3SS in *D. dadantii*, we examined the expression of *hrp* genes, including *hrpA* and *hrpN*, in the *gcpD* deletion mutant. As shown in Fig. 1A, significant increases in *hrpA* and *hrpN* promoter activities were observed in ∆*gcpD* relative to wild type, and complementation of *gcpD* with its native promoter in a low copy number plasmid (pCL1920-*gcpD*) restored the phenotype.

To assess the underlying mechanism of GcpD-mediated T3SS regulation, the transcriptional activities of several T3SS regulatory genes were determined upon the deletion of *gcpD*. A significant increase in the transcription of *hrpL* and *hrpS* was observed (Fig. 1A), while the *rpoN* promoter activity was not altered (Fig. S1). We also measured the RNA levels of *rpoN* via RT-qPCR and found that deletion of *gcpD* increased the RNA levels of *rpoN* by approximately threefold (Fig. 1B). Collectively, these results suggest that GcpD likely regulates the expression of T3SS genes by post-transcriptionally modulating RpoN, which subsequently controls the T3SS master regulatory HrpL.

### The DGC activity of GcpD is essential for the regulation of T3SS

**Fig 2:**
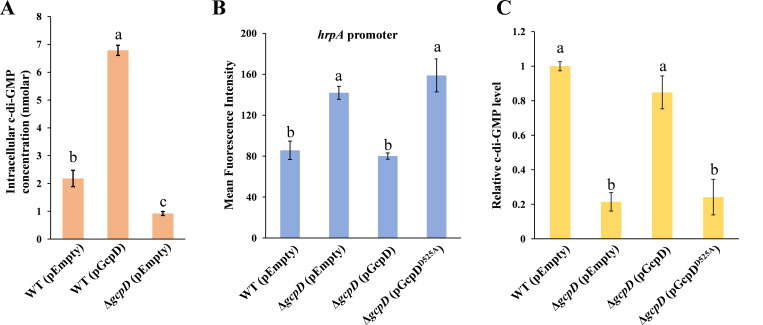
The DGC activity of GcpD is essential for T3SS regulation. (**A**) Measurement of intracellular c-di-GMP using ultraperformance liquid chromatography coupled with tandem mass spectrometry in wild-type (WT) *D. dadantii* harboring empty vector pCL1920, WT bacteria harboring pCL1920-*gcpD*, and Δ*gcpD* harboring pCL1920. (**B**) *hrpA* promoter activities were measured in WT *D. dadantii* harboring pCL1920, Δ*gcpD* harboring pCL1920, Δ*gcpD* harboring pCL1920-*gcpD*, and Δ*gcpD* harboring pCL1920-*gcpD*^D525A^, respectively. (**C**) Intracellular c-di-GMP level calculated by *β*-galactosidase activity from a c-di-GMP riboswitch fusion using pRS414 vector in WT *D. dadantii* harboring pCL1920, Δ*gcpD* harboring pCL1920, Δ*gcpD* harboring pCL1920-*gcpD*, and Δ*gcpD* harboring pCL1920-*gcpD*^D525A^, respectively. Three independent experiments were performed, and three replicates were used for each experiment. Error bars indicate standard errors of the means. The lowercase letters above the bars indicate statistically significant differences between treatments (*P* < 0.05) by one-way ANOVA.

As mutation of *gcpD* enhanced the expression of T3SS genes, we hypothesized that such regulation relies on c-di-GMP signaling. To address this, the intracellular concentrations of c-di-GMP were measured in WT *D. dadantii*, ∆*gcpD*, and WT overexpressing *gcpD* using ultra-performance liquid chromatography combined with tandem mass spectrometry (UPLC-MS/MS). Our result showed that the c-di-GMP concentration in *gcpD* overexpressing strain was approximately threefold higher than in WT harboring an empty vector (Fig. 2A). Furthermore, the c-di-GMP levels in ∆*gcpD* were significantly lower than wild type (Fig. 2A). Taken together, these results suggest that GcpD is indeed a DGC that synthesizes c-di-GMP in *D. dadantii*.

To test our hypothesis that the c-di-GMP-producing activity of GcpD is crucial for T3SS regulation, we introduced a single amino acid substitution, replacing the critical aspartic acid residue with alanine (AGDEF to AGAEF) in the GGDEF domain of GcpD. This motif, also known as active site (A-site), is responsible for the synthesis of c-di-GMP from GTP (31, 32). As expected, alteration of the A-site (*gcpD*^D525A^) failed to restore *hrpA* promoter activity in ∆*gcpD* (Fig. 2B). Meanwhile, intracellular c-di-GMP levels were consistent with the transcriptional analysis, showing that, unlike the WT *gcpD*, the expression of *gcpD*^D525A^ did not restore c-di-GMP levels in the ∆*gcpD* mutant to those of the wild type (Fig. 2C).

### Transposon mutagenesis identified CdeR as a T3SS regulator

**Fig 3:**
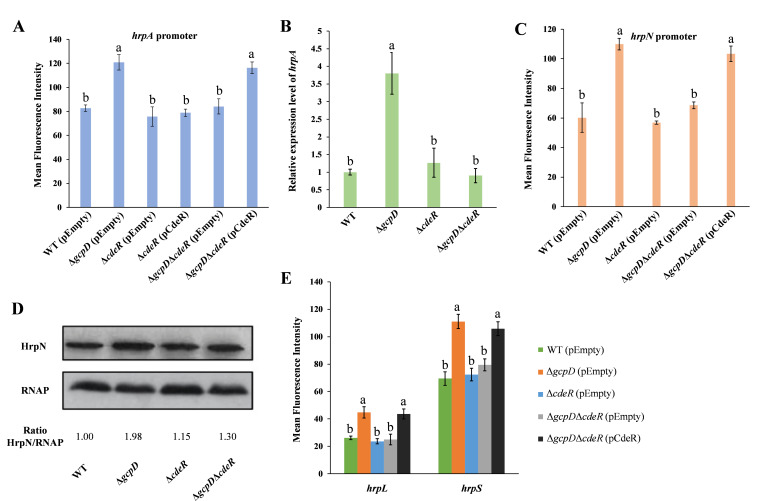
CdeR is involved in T3SS regulation. (**A**) Promoter activities of *hrpA* were determined in wild-type (WT) *D. dadantii* harboring the empty vector pCL1920, Δ*gcpD* harboring pCL1920, Δ*cdeR* harboring pCL1920, Δ*cdeR* harboring pCL1920-*cdeR*, Δ*gcpD*Δ*cdeR* harboring pCL1920, and Δ*gcpD*Δ*cdeR* harboring pCL1920-*cdeR*. (**B**) RNA levels of *hrpA* were measured in wild type, Δ*gcpD*, Δ*cdeR*, and Δ*gcpD*Δ*cdeR*. (**C**) Promoter activities of *hrpN* were measured in WT bacteria harboring pCL1920, Δ*gcpD* harboring pCL1920, Δ*cdeR* harboring pCL1920, Δ*gcpD*Δ*cdeR* harboring pCL1920, and Δ*gcpD*Δ*cdeR* harboring pCL1920-*cdeR*. (**D**) Western blot analysis of HrpN protein levels in WT *D. dadantii*, Δ*gcpD*, Δ*cdeR,* and Δ*gcpD*Δ*cdeR*. (**E**) The T3SS regulon genes *hrpL* and *hrpS* promoter activities were examined in the above-mentioned strains. Values are representative of three independent experiments. Three replicates were used in each experiment. The lowercase letters above the bars indicate statistically significant differences between treatments (*P* < 0.05) by one-way ANOVA.

To understand how GcpD controls the expression of T3SS in *D. dadantii*, we conducted a transposon mutagenesis screen in ∆*gcpD* carrying a reporter plasmid pProbeAT*-hrpA*. We found that the insertion of the MiniHimar RB1 transposon in the open reading frame of ABF-0020041, encoding a putative DNA binding transcription regulator, significantly decreased *hrpA* expression. We then named ABF-0020041 as CdeR (c-di-GMP dependent regulator). To validate our transposon mutagenesis analysis, *cdeR* was chromosomally deleted in WT and Δ*gcpD* backgrounds, respectively. Although no significant difference in the *hrpA* promoter activity was detected between wild type and Δ*cdeR*, the promoter activity and RNA levels of *hrpA* were decreased in *∆gcpD∆cdeR* relative to Δ*gcpD* (Fig. 3A and B). Complementation of *cdeR* in *∆gcpD∆cdeR* restored the phenotype to WT levels (Fig. 3A). Furthermore, we observed that the *hrpN* promoter activity and protein levels were significantly decreased in Δ*gcpD*Δ*cdeR* compared to Δ*gcpD* (Fig. 3C and D).

Next, we examined the promoter activities of *hrpL*, *hrpS*, and *rpoN* in wild-type, Δ*gcpD*, Δ*cdeR*, and *∆gcpD∆cdeR* strains. A decrease in *hrpL* and *hrpS* promoter activity was observed in *∆gcpD∆cdeR* relative to Δ*gcpD* (Fig. 3E), while similar *rpoN* promoter activities and RNA levels were detected between Δ*gcpD* and *∆gcpD∆cdeR* (Fig. S2). Additionally, deletion of *cdeR* alone did not affect the expression of T3SS regulatory genes (Fig. 3E; Fig. S2). Taken together, these results suggest that CdeR positively regulates the T3SS only in the absence of *gcpD*, involving HrpL and HrpS but not RpoN.

### CdeR regulates the T3SS in Δ*gcpD* by manipulating c-di-GMP levels

**Fig 4:**
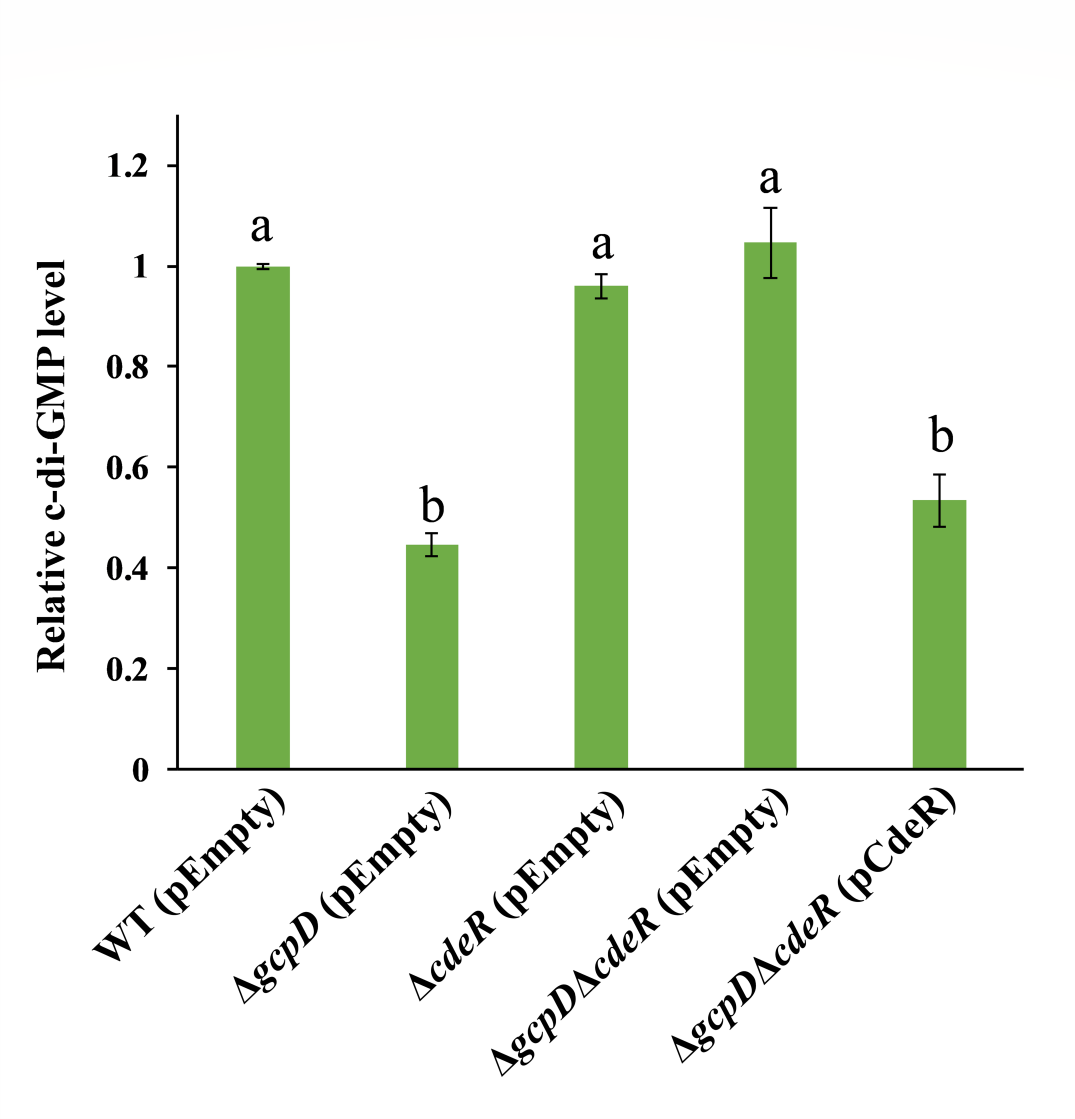
Deletion of *cdeR* in Δ*gcpD* restores c-di-GMP levels. Relative c-di-GMP levels were examined in wild-type (WT) *D. dadantii* harboring the empty vector pCL1920, Δ*gcpD* harboring pCL1920, Δ*cdeR* harboring pCL1920, Δ*gcpD*Δ*cdeR* harboring pCL1920, and Δ*gcpD*Δ*cdeR* harboring pCL1920-*cdeR*. The data represent GUS values of each gene relative to the WT. The calculation was made by measuring the *β*-galactosidase activity from a c-di-GMP riboswitch fusion using the pRS414 vector. The lowercase letters above the bars indicate statistically significant differences between treatments (*P* < 0.05) by one-way ANOVA.

To assess how c-di-GMP signaling plays a role in CdeR-mediated T3SS regulation, we measured the intracellular c-di-GMP levels in ∆*cdeR*∆*gcpD* using a c-di-GMP reporter that harbors a transcriptional fusion of *β*-glucuronidase to a 110-nucleotide Vc2 RNA riboswitch (33). Interestingly, our result revealed that the deletion of *cdeR* in ∆*gcpD* restored its c-di-GMP to WT levels, which could be complemented by in *trans* expression of *cdeR* (Fig. 4). In addition, the deletion of *cdeR* alone did not affect intracellular c-di-GMP levels (Fig. 4).

**Fig 5:**
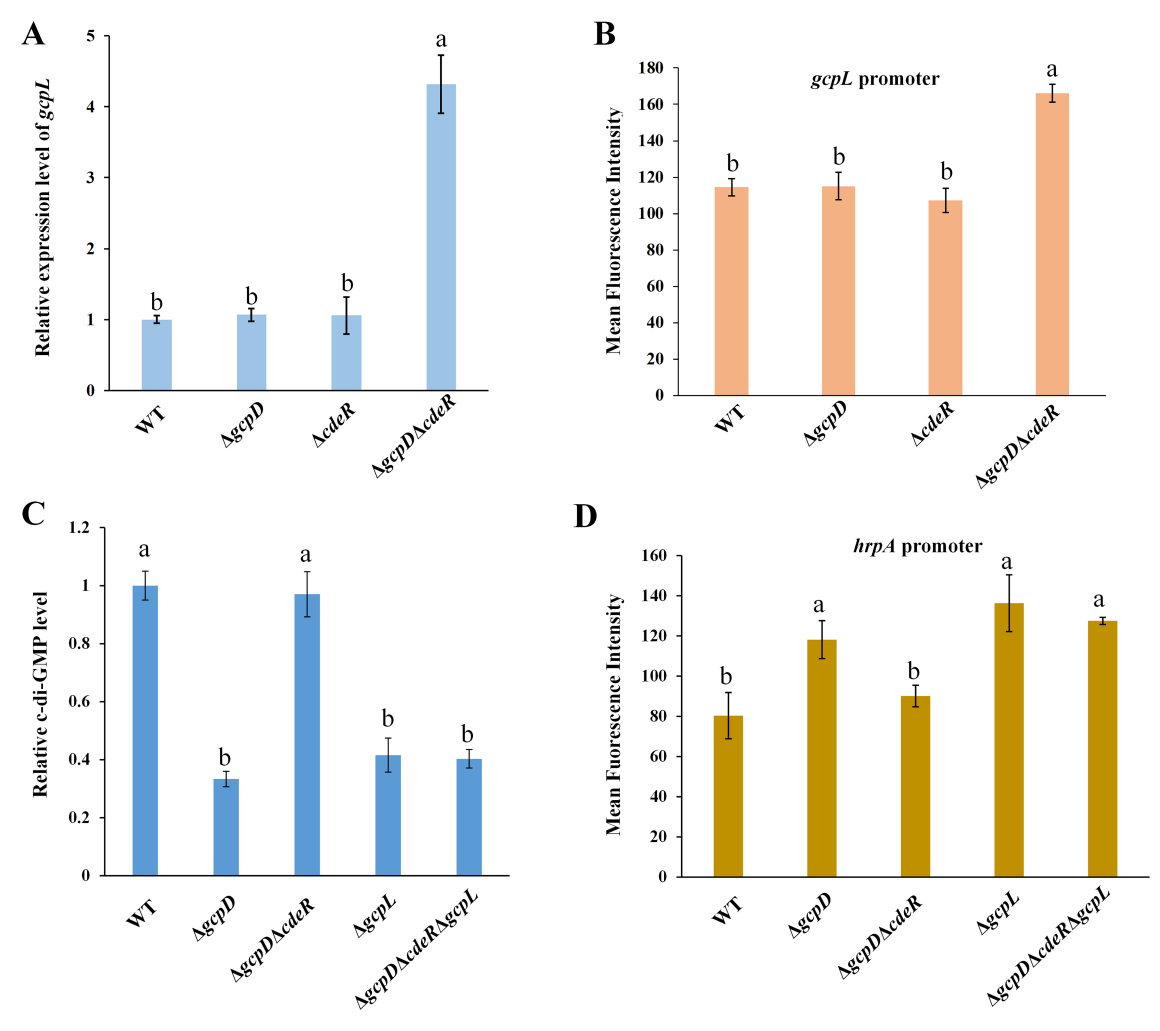
CdeR-mediated T3SS regulation relies on GcpL. (**A**) RNA levels and (**B**) promoter activities of *gcpL* were examined in wild-type (WT) *D. dadantii*, Δ*gcpD*, Δ*cdeR*, and Δ*gcpD*Δ*cdeR*. (**C**) Relative c-di-GMP concentrations were examined in WT *D. dadantii*, Δ*gcpD*, Δ*gcpD*Δ*cdeR*, Δ*gcpL*, and Δ*gcpD*Δ*cdeR*Δ*gcpL*. (**D**) *hrpA* promoter activities were measured in the above-mentioned strains. Similar results were observed in three independent experiments with three technical replicates. The lowercase letters represent different treatment groups with significant statistical differences, whereas treatments with no significant differences were shown the same letters (*P* < 0.05) by one-way ANOVA.

To identify which DGCs are responsible for the increased c-di-GMP levels in *∆gcpD∆cdeR*, we measured the RNA levels of *gcpL*, a known DGC-encoding gene (Fig. S3), using RT-qPCR. As a result, a fourfold increase in *gcpL* RNA levels was detected in *∆gcpD∆cdeR* compared to *∆gcpD* (Fig. 5A), suggesting that CdeR represses *gcpL* expression. To determine the level at which CdeR regulates GcpL, we examined its promoter activity in WT and mutant bacteria. Increased promoter activity of *gcpL* was observed in *∆gcpD∆cdeR* relative to ∆*gcpD*, comparable to the WT strain (Fig. 5B). Notably, the deletion of *gcpL* in *∆gcpD∆cdeR* resulted in decreased intracellular c-di-GMP levels and increased *hrpA* promoter activity (Fig. 5C and D). These findings suggest that CdeR positively regulates the T3SS by suppressing *gcpL* expression. Since *∆cdeR* did not exhibit any effects on the transcription or post-transcription of *gcpL* (Fig. 5A and B), we hypothesize that low c-di-GMP levels are likely needed for the regulation of GcpL by CdeR. To validate this, a PDE-encoding gene, *egcpB*, was overexpressed in ∆*cdeR*∆*gcpD* and found that c-di-GMP levels and *hrpA* expression were restored to near ∆*gcpD* levels (Fig. S4).

### CdeR controls bacterial motility, *pelD* gene expression, and potato maceration in a c-di-GMP-dependent manner

**Fig 6:**
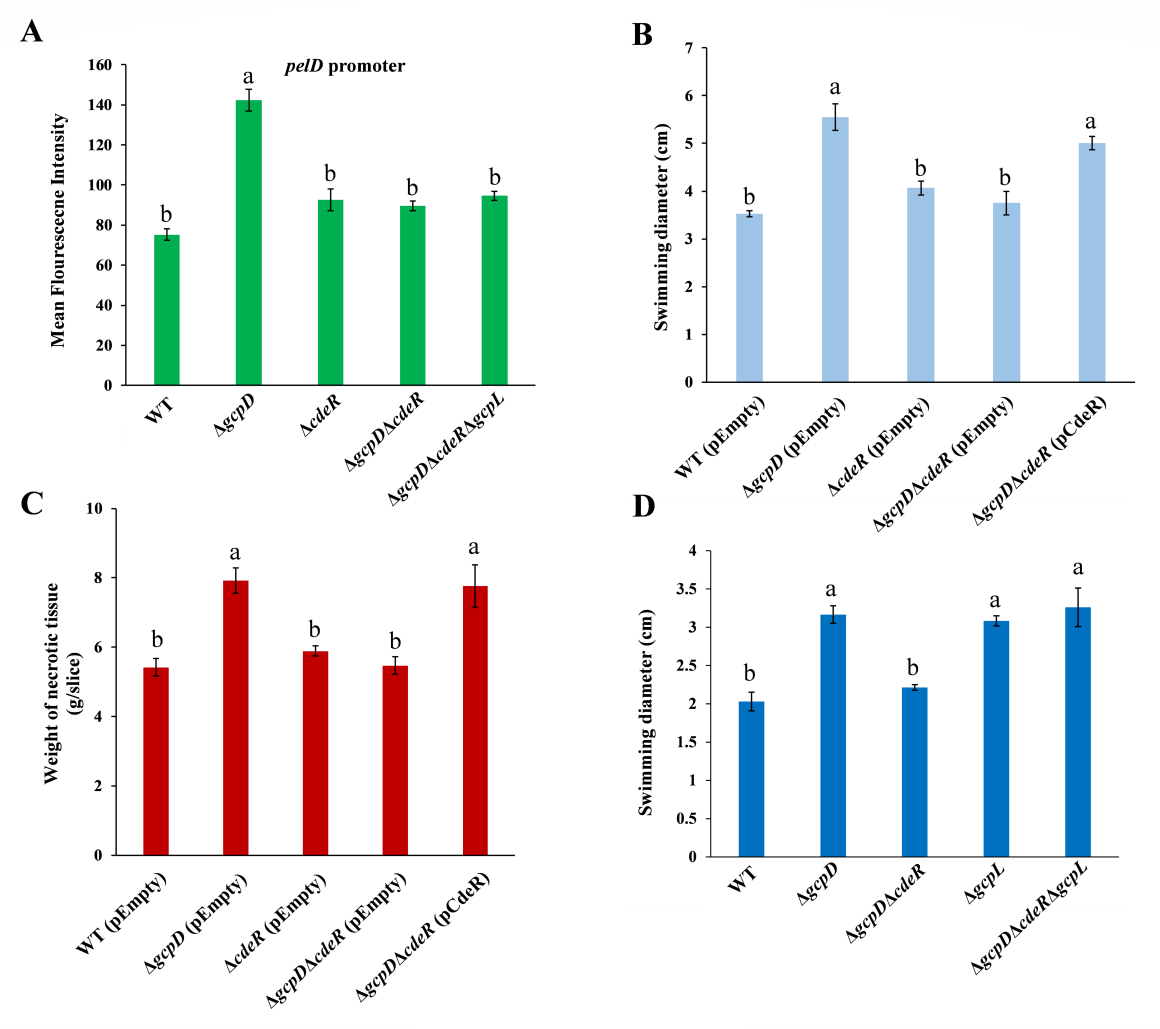
CdeR is involved in the regulation of *pelD* expression, swimming motility, and virulence. (**A**) *pelD* promoter activities were measured in *D. dadantii* wild type, Δ*gcpD,* Δ*cdeR,* Δ*gcpD*Δ*cdeR*, and Δ*gcpD*Δ*cdeR*Δ*gcpL*. (**B**) The swimming motility diameter was examined in wild-type (WT) *D. dadantii* harboring the empty vector pCL1920, Δ*gcpD* harboring pCL1920, Δ*cdeR* harboring pCL1920, Δ*gcpD*Δ*cdeR* harboring pCL1920, and Δ*gcpD*Δ*cdeR* harboring pCL1920-*cdeR*. (**C**) The weight of necrotic tissue was measured after the inoculation of WT *D. dadantii* harboring pCL1920, Δ*gcpD* harboring pCL1920, Δ*cdeR* harboring pCL1920, Δ*gcpD*Δ*cdeR* harboring pCL1920, and Δ*gcpD*Δ*cdeR* harboring pCL1920-*cdeR*. (**D**) The swimming motility diameter was measured in *D. dadantii* wild type, Δ*gcpD,* Δ*gcpD*Δ*cdeR*, Δ*gcpL,* and Δ*gcpD*Δ*cdeR*Δ*gcpL*. Values are representative of three experiments, and three replicates were used for each experiment. Error bars indicate standard errors of the means. The lowercase letters indicate statistically significant differences between treatments (*P* < 0.05) by one-way ANOVA.

To explore whether CdeR controls additional c-di-GMP-associated phenotypes and virulence beyond the T3SS, we tested bacterial motility, the expression of pectate lyase encoding gene *pelD*, and potato maceration in mutant strains lacking *cdeR*. As shown in Fig. 6A, a significant decrease in *pelD* promoter activity was observed in ∆*gcpD∆cdeR* compared to *∆gcpD*, whereas deletion of *cdeR* alone in WT bacteria had negligible impact. This result suggests that CdeR positively regulates *pelD* gene expression only when *gcpD* is deleted. Furthermore, deletion of *gcpD* led to enhanced swimming motility and an increased maceration area on potato tubers, both of which were restored to WT levels upon deletion of *cdeR* (∆*gcpD*∆*cdeR*) (Fig. 6B and C). Complementation of *cdeR* in ∆*gcpD*∆*cdeR* recovered the phenotypes to ∆*gcpD* levels (Fig. 6B and C). Similar to the regulation of the *pelD* gene, the deletion of *cdeR* alone did not affect motility or virulence in potatoes (Fig. 6B and C).

Since we observed that GcpL is crucial for the regulation of T3SS by CdeR, we next examined the *pelD* expression and swimming motility in the *∆gcpD∆cdeR*∆*gcpL* triple deletion mutant. Our results revealed that deleting *gcpL* increased the swimming motility of *∆gcpD∆cdeR* to levels comparable to *∆gcpD* (Fig. 6D), but it did not affect *pelD* expression (Fig. 6A). This is worth noting because GcpL itself did not regulate Pel (34), yet its deletion in WT *D. dadantii* led to enhanced swimming (Fig. 6A and D). In summary, these results indicate that CdeR controls bacterial motility by modulating c-di-GMP levels through GcpL, while its control of the *pelD* gene expression is not involving GcpL.

### The DNA-binding domain of CdeR is essential for its function

**Fig 7:**
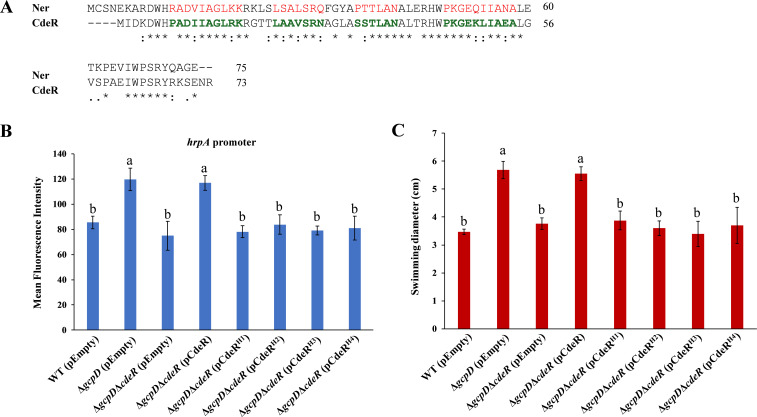
The HTH region of CdeR is cortical for its function. (**A**) Sequence alignments of CdeR and Ner protein. Amino acids highlighted in red show the HTH regions in Ner. Amino acids highlighted in green are putative HTH regions in CdeR. “*” indicates identical amino acid, “:” indicates conserved substitution, “.” indicates semi-conserved substitution, and no label indicates non-conserved substitution. (**B**) *hrpA* promoter activities and (**C**) swimming motilities were determined in wild-type *D. dadantii* harboring the empty vector pCL1920, Δ*gcpD* harboring pCL1920, Δ*gcpD*Δ*cdeR* harboring pCL1920, and Δ*gcpD*Δ*cdeR* harboring pCL1920-*cdeR*, Δ*gcpD*Δ*cdeR* harboring pCL1920-*cdeR*^H1^, Δ*gcpD*Δ*cdeR* harboring pCL1920-*cdeR*^H2^, Δ*gcpD*Δ*cdeR* harboring pCL1920-*cdeR*^H3^, and Δ*gcpD*Δ*cdeR* harboring pCL1920-*cdeR*^H4^. The lowercase letters indicate statistically significant differences between treatments (*P* < 0.05) by one-way ANOVA.

Sequence alignment analysis revealed that CdeR shares approximately 59% amino acid sequence identity with the Ner protein, a known DNA-binding protein in phage Mu, and 64% identity with the Nlp protein in *Escherichia coli* (35, 36). All of these proteins contain an HTH DNA-binding domain. To test whether the *D. dadantii* CdeR functions as a DNA-binding protein, we investigated four potential helical regions: Pro8–Lys17 (H1), Leu22–Asn28 (H2), Ser33–Asn38 (H3), and Pro45–Ala54 (H4), for their role in CdeR-mediated regulation of T3SS and motility (Fig. 7A). Previous research has shown that helices H2 and H3 form an HTH motif in the Ner protein to interact with DNA, while other helices provide structural support (36). As a result, CdeR derivatives lacking these helical regions failed to increase the expression of *hrpA* gene in *∆gcpD∆cdeR* (Fig. 7B), and similar outcomes were observed for swimming motility (Fig. 7C). Additionally, the stability of CdeR derivatives was confirmed (Fig. S5). These data suggest that CdeR acts as a potential DNA-binding protein, and the HTH regions are necessary for its biological function.

## DISCUSSION

In this study, we first demonstrated that the DGC GcpD inhibits the expression of T3SS genes in a c-di-GMP-dependent manner in *D. dadantii*. We further revealed that GcpD controls the T3SS likely through the RpoN-HrpS-HrpL pathway, as the *rpoN* mRNA levels and *hrpS* promoter activity were significantly increased in *∆gcpD* compared to the wild type. Transposon mutagenesis identified CdeR as a crucial factor, with its deletion restoring T3SS phenotypes to WT levels in *∆gcpD*. Interestingly, CdeR regulated the T3SS in *D. dadantii* only under *gcpD* mutant background, a process that appears to involve another DGC, GcpL, and is likely independent of RpoN. Similar patterns were observed for other known c-di-GMP-mediated behaviors and virulence factors, including swimming motility and Pel production. CdeR was recognized as a putative transcriptional regulator conserved among soft rot bacteria, including *D. zeae* (98% amino acid identity), *D. dianthicola* (98%), and *Pectobacterium* (97%). Our results demonstrated that the DNA-binding domain of CdeR is crucial for its biological function in *D. dadantii*.

The bacterial second messenger c-di-GMP inhibits the expression of T3SS genes in *D. dadantii* (14). Two PDEs, EGcpB and EcpC, have been shown to positively regulate the master regulator HrpL via RpoN at the post-transcriptional level, but the transcriptional level of *hrpS* was not affected (14). Among DGCs, a previous study demonstrated that GcpA, whose deletion mutant showed elevated T3SS gene expression likely through increased *rsmB* levels, functions independently of RpoN (15). Interestingly, we observed in this study that the regulatory mechanism of GcpD on T3SS involves both HrpS and RpoN at transcriptional and post-transcriptional levels, respectively. Taken together, these results suggest a hierarchical regulation of different c-di-GMP signaling pathways controlling the T3SS in *D. dadantii*. GcpD likely pairs with EGcpB and EcpC to transcriptionally regulate HrpL, while GcpA fine-tunes the stability of *hrpL* mRNA by modulating the RsmA/*rsmB* system. It is worth noting that EGcpB and EcpC are independently involved in the regulation of RsmB during the production of cell-wall-degrading enzymes; however, this regulation was not observed under T3SS-inducing conditions (15).

Our transposon mutagenesis identified CdeR as a key player in GcpD-mediated regulation in *D. dadantii*. Interestingly, the deletion of *cdeR* alone had a negligible impact on several c-di-GMP-associated cellular behaviors and virulence, suggesting that CdeR exerts its function only in the absence of GcpD. This is not new to the c-di-GMP signaling in bacteria. For example, in *Pseudomonas aeruginosa*, the transcriptional regulator FleQ controls both flagellar motility (at low c-di-GMP levels) and biofilm formation (at high levels). In the absence of c-di-GMP, FleQ acts as an activator of flagellar assembly genes. However, when it binds to c-di-GMP, FleQ represses motility and switches its function to activate genes responsible for biofilm formation, such as the exopolysaccharide synthesis genes (37–39). In *D. dadantii*, we demonstrated that the c-di-GMP effector BcsA controls biofilm formation and Pel production when the c-di-GMP levels are high (16). Given the low intracellular c-di-GMP levels in ∆*gcpD*, our results indicate that CdeR is likely inactive under WT or high c-di-GMP level conditions; however, it remains unclear whether GcpD is the sole DGC involved. Other intriguing questions remain regarding the potential interplay between GcpD, CdeR, and c-di-GMP. Specifically, it remains unclear whether CdeR directly binds c-di-GMP, a phenomenon observed in transcriptional regulators across various bacterial species (38, 40–46). Additionally, the possibility of a direct interaction between GcpD and CdeR has yet to be explored. In *E. coli*, the DGC YdaM has been found to activate the transcriptional factor MlrA through direct interaction (47). Moreover, physical interactions between transcriptional regulators and effectors have been widely reported (48, 49), providing specificity to c-di-GMP signaling pathways.

Despite our observation that CdeR is crucial for T3SS regulation, how it controls the expression of T3SS regulon genes remains unclear. A previous study demonstrated that SlyA, a transcriptional regulator, positively controls *hrpA* transcription in *D. dadantii* (50). SlyA has also been reported to regulate Pel production and overall virulence *in planta* (29). We observed that both the promoter activity and RNA levels of *slyA* decreased in *∆gcpD* and were restored to the WT level in *∆gcpD∆cdeR* (data not shown). This result suggests that SlyA is unlikely to play a key role in CdeR-mediated T3SS regulation, as a decreased, rather than increased, *slyA* expression would be expected in *∆gcpD∆cdeR*. We did, however, highlight the importance of the HTH region for CdeR’s biological functions. Expression of the HTH-deleted *cdeR* in Δ*gcpD*Δ*cdeR* failed to restore T3SS expression and swimming motility compared to WT *cdeR*. It remains to be elucidated whether CdeR directly interacts with the T3SS regulon, and further investigation will be conducted to identify putative c-di-GMP-associated regulators.

**Fig 8:**
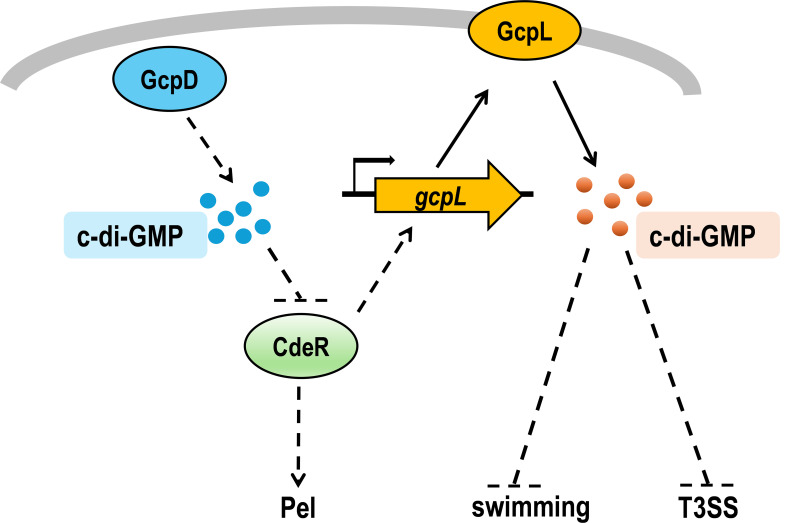
Proposed model of CdeR-mediated regulation in *D. dadantii*. Two DGCs, GcpD and GcpL, produce c-di-GMP. GcpD is likely a cytoplasmic protein while GcpL is a membrane protein. GcpD-produced c-di-GMP inhibits the function of CdeR via an unknown mechanism. CdeR positively regulates *gcpL* transcription and Pel gene expression. GcpL-produced c-di-GMP negatively regulates T3SS gene expression and bacterial swimming motility in *D. dadantii*. Arrows and bars represent activation and repression, respectively. The dotted lines represent regulations found in this study.

Interestingly, we found that c-di-GMP levels in *∆gcpD∆cdeR* were restored to WT levels, due to increased expression of *gcpL*, which encodes a DGC in *D. dadantii* (17). Deletion of *gcpL* in *∆gcpD∆cdeR* lowered its c-di-GMP levels, leading to increased *hrpA* promoter activities and swimming motility. These observations suggest the involvement of two distinct c-di-GMP signaling pathways that control the T3SS and motility in *D. dadantii* (Fig. 8). During the repression of T3SS and swimming motility, the DGC GcpD synthesizes c-di-GMP, which inactivates CdeR. As c-di-GMP is degraded, CdeR inhibition is alleviated, resulting in the repression of *gcpL* transcription. GcpL produces c-di-GMP to negatively regulate T3SS and swimming, although these c-di-GMP molecules do not appear to affect the inactivation of CdeR. The specific PDEs involved in balancing c-di-GMP synthesis and degradation remain unknown, as well as environmental signals or intracellular cues that influence c-di-GMP metabolism. Differing from the regulation of T3SS and swimming motility, the deletion of *gcpL* in *∆gcpD∆cdeR* did not restore its *pelD* gene expression. This result suggests that CdeR regulates *pelD* expression through an unknown mechanism independent of GcpL.

In summary, our study demonstrated the positive role of CdeR on T3SS gene expression under low c-di-GMP level conditions. When the expression of T3SS is not required, *D. dadantii* cells synthesize c-di-GMP via the DGC enzymes to inactivate CdeR. We also proposed a model illustrating the crosstalk between CdeR and c-di-GMP signaling in the regulation of T3SS, swimming motility, and Pel production in *D. dadantii* (Fig. 8). Understanding these interactions provides a comprehensive view of how bacterial cells adapt their behaviors and regulate virulence in response to environmental changes.

## MATERIALS AND METHODS

### Bacterial strains, plasmids, primers, and culture

The bacterial strains and plasmids used in this study are listed in Table 1. The primer information is listed in Table S1. *D. dadantii* strains were cultured in lysogeny broth (LB) (1% tryptone, 0.5% yeast extract, and 1% NaCl), mannitol-glutamic acid (MG) medium (1% mannitol, 0.2% glutamic acid, 0.05% potassium phosphate monobasic, 0.02% MgSO_4_, and 0.02% NaCl) or low nutrient T3SS-inducing minimal medium (MM) (0.6% Na_2_HPO_4_, 0.3% KH_2_PO_4_, 0.1% NH_4_Cl, 0.05% NaCl, 2% glycerol, 1 mM MgSO_4_, and 0.1 mM CaCl_2_) at 28°C (51). *E. coli* strains were grown in LB medium at 37°C. Antibiotics were added at the stated concentrations: ampicillin (100 µg mL^−1^), kanamycin (50 µg mL^−1^), and spectinomycin (100 µg mL^−1^). The genomic sequence of *D. dadantii* was obtained from the Annotated Systematic Annotation Package (ASAP) for community genome analysis (https://asap.genetics.wisc.edu/asap/home.php).

**TABLE 1:** List of strains and plasmids

Strains and plasmids	Relevant characteristics^*a*^	Reference or source
*Dickeya dadantii*		
3937	Wild-type	Lab stock
Δ*gcpD*	Δ*gcpD*::*Km*; Km^r^, ABF-0014719 deletion mutant	(15)
Δ*gcpD*^D525A^	Δ*gcpD*^D525A^::*Km*; Km^r^, ABF-0014719 site-directed mutant	This study
Δ*cdeR*	Δ*cdeR*::*Km*; Km^r^, ABF-0020041 deletion mutant	This study
Δ*gcpD*Δ*cdeR*	Δ*gcpD*Δ*cdeR*::*Km*; Km^r^, ABF-0014719 and ABF-0020041 double deletion mutant	This study
Δ*gcpL*	Δ*gcpL*::*Km*; Km^r^, ABF-0015843 deletion mutant	This study
Δ*gcpD*Δ*cdeR*Δ*gcpL*	Δ*gcpD*Δ*cdeR*Δ*gcpL*::*Km*; Km^r^, ABF-0014719, ABF-0020041, and ABF-0015843 triple deletion mutant	This study
∆*egcpB*	Δ*egcpB::Km*; Km^r^, ABF-0020123 deletion mutant	(14)
*Escherichia coli*		
DH5α	*supE*44 Δ*lac*U169 (*ϕ*80*lacZ*ΔM15) *hsdR*17 *recA*1 *endA*1 *gyrA*96 *thi*-1 *relA*1	Lab stock
S17-1 λpir	λ(pir) *hsdR* pro *thi*; chromosomally integrated RP4-2 Tc::Mu Km::Tn7	Lab stock
Plasmids		
pKD4	Template plasmid for kanamycin cassette, Km^r^	(52)
pWM91	Sucrose-based counter-selectable plasmid, Ap^r^	(53)
pFLP2	Flipase FRT excision vector, Ap^r^	(54)
pWM91-*vfmH*	pWM91 harboring flanking regions of *vfmH* with kanamycin cassette in between, Km^r^, Ap^r^	This study
pCL1920	Low copy number plasmid, lac promoter, Sp^r^	(55)
pCL1920-*cdeR*	*cdeR* cloned in pCL1920 under *lac* promoter, Sp^r^	This study
pCL1920-*gcpD*	*gcpD* cloned in pCL1920 under *lac* promoter, Sp^r^	This study
pCL1920-*hrpL*	*hrpL* cloned in pCL1920 under *lac* promoter, Sp^r^	This study
pCL1920-*hrpS*	*hrpS* cloned in pCL1920 under *lac* promoter, Sp^r^	This study
pCL1920-*gcpL*	*gcpL* cloned in pCL1920 under *lac* promoter, Sp^r^	This study
pPROBE-AT	Promoter-probe vector, promoter-less *gfp*, Ap^r^	(56)
pAT-*pelD*	pPROBE-AT containing putative *pelD* promoter-*gfp* transcriptional fusion, Ap^r^	(57)
pAT-*hrpA*	pPROBE-AT containing putative *hrpA* promoter-*gfp* transcriptional fusion, Ap^r^	(58)
pAT-*hrpN*	pPROBE-AT containing putative *hrpN* promoter-*gfp* transcriptional fusion, Ap^r^	(58)
pAT-*hrpS*	pPROBE-AT containing putative *hrpS* promoter-*gfp* transcriptional fusion, Ap^r^	(58)
pAT-*hrpL*	pPROBE-AT containing putative *hrpL* promoter-*gfp* transcriptional fusion, Ap^r^	(58)
pAT-*gcpL*	pPROBE-AT containing putative *gcpL* promoter-*gfp* transcriptional fusion, Ap^r^	This study
pAT-*slyA*	pPROBE-AT containing putative *slyA* promoter-*gfp* transcriptional fusion, Ap^r^	(50)

^*a*^ Ap^r^, ampicillin resistance; Km^r^, kanamycin resistance; Sp^r^, spectinomycin resistance.

### Mutant construction and complementation

∆*cdeR* was constructed by marker exchange mutagenesis (59). Briefly, upstream and downstream fragments flanking the *cdeR* gene were amplified by PCR with specific primers (Table S1). The kanamycin cassette (~1.5 kb) was amplified from the pKD4 plasmid (52) and cloned between flanking regions performing three-way crossover PCR. The PCR construct was ligated into the suicide plasmid pWM91 and transformed into *D. dadantii* cells by conjugation with *E. coli* strain S17-1 λ-pir. To select strains with chromosomal deletions, kanamycin-resistant recombinants were plated onto MG plus sucrose (0.1 g/mL) agar plate. The cells that were resistant to sucrose because of the loss of SacB-mediated toxicity were then plated onto LB-containing ampicillin and LB-containing kanamycin plates separately. Mutants sensitive to ampicillin were picked and confirmed by PCR using outside primers and sequencing.

To construct double and triple mutants, the pFLP2 plasmid encoding the FLP (flipase) recombinase enzyme in *E. coli* S17-1 λ-pir was conjugated with mutants harboring kanamycin cassette. Two FLP recombinase target (FRT) sites flanking the kanamycin cassette enabled the excision through flipase. Transconjugants resistant to sucrose and sensitive to kanamycin were selected and confirmed by outside primer and sequencing. The double and triple mutants were constructed similarly. To construct complemented strains, the promoter and open reading frame region of target genes were PCR amplified and inserted into low copy number plasmid pCL1920 (Table 1).

### GFP reporter plasmid construction and flow cytometry assay

To generate the reporter plasmids pAT*-gcpL* and pAT*-cdeR*, the promoter regions and a small portion of open reading frames of target genes were PCR amplified and cloned into the promoter probe vector pPROBE-AT, a vector that contains promoterless *gfp*, whose expression is driven by promoter cloned upstream (60). The reporter plasmids pAT *-hrpA*, pAT *-hrpN,* pAT *-hrpL*, pAT *-dspE,* pAT *-slyA*, and pAT *-pelD* were constructed previously following the same procedure (Table 1). Bacterial cells harboring the reporter plasmids were grown in LB broth overnight and transferred to MM broth in a 1:100 ratio. Samples were collected in 16 hours and the mean fluorescence intensity (MFI) of each sample was monitored by flow cytometry (BD Biosciences, San Jose, CA, USA) standardized by FACS Caliber (57). The *pelD* promoter activity was measured with bacterial cultures grown in MM broth supplemented with 0.1% polygalacturonic acid (PGA).

### Western blot analysis

*D. dadantii* cells grown in MM broth for 12 h were resuspended in phosphate-buffered saline (PBS) and lysed by sonication. The protein in crude lysates was quantified using the Bradford protein assay (Bio-Rad, Hercules, CA, USA). Samples were boiled before loading onto 12% sodium dodecyl sulfate polyacrylamide gels. Proteins were transferred onto a PVDF membrane (Millipore, Bedford, MA, USA). Blots were washed with PBS containing 0.05% Tween-20 and probed with anti-HrpN antibodies (Proteintech, Rosemont, IL, USA). Anti-RNA polymerase monoclonal antibody (Neoclone, Madison, WI, USA) was used as a control. The primary antibodies were used at a 1:1,000 dilution and incubated with the membrane overnight at 4°C. Anti-Mouse IgG-HRP conjugate secondary antibody (Southern Biotech, Birmingham, AL, USA) was used at a 1:5,000 dilution, with a 1-h incubation at room temperature. The resulting blots were incubated for 1 min in enhanced chemiluminescence reagent (GE Healthcare, Chicago, IL, USA) and detected using an O-MAT X-ray film.

### Reverse transcription (RT) and quantitative RT-PCR (RT-qPCR) analysis

A reverse transcriptase PCR assay was performed to detect the RNA level of *egcpB*, *ecpC*, *egcpA*, *gcpA*, *gcpL*, *gcpD*, along with *hrpA*, *cdeR*, and *slyA*. The mRNA levels of *hrpA*, *slyA*, and *gcpL* were measured by qRT-PCR. Briefly, bacterial cells cultured in MM for 12 h were harvested, and the total RNA from the bacteria was isolated by using TRI (Sigma-Aldrich, St. Louis, USA) reagent method, followed by phenol/chloroform extraction and isopropanol precipitation. DNase treatment with gDNA Wipeout Buffer (QIAGEN, Hilden, Germany) was performed according to the manufacturer’s instructions. cDNA was synthesized by preparing a Reverse Transcription master mix (QIAGEN, Hilden, Germany). The iQ SYBR Green Supermix (Bio-Rad, Hercules, CA, USA) was used for the qRT-PCR reaction to quantify the cDNA levels of each target gene. The *rplU* was used as the endogenous control to normalize the cDNA input of each sample. Data were collected by the Opticon 2 system (Bio-Rad, Hercules, CA, USA) and analyzed using the 2^–∆∆CT^ method (61).

### Determination of the intracellular c-di-GMP concentration

Intracellular c-di-GMP concentrations were determined using UPLC-MS/MS as described previously (62). Briefly, overnight bacterial cultures were inoculated 1:100 into 50 mL LB broth in a flask. After the optical density values at 600 nm (OD_600_) of bacterial cultures reached around 0.8, corresponding to mid-to-late-exponential growth, the cells were centrifuged for 30 min at 4,000 rpm. Then, the supernatant was removed, and the pellet was resuspended in a cold 1.5 mL extraction buffer (40% acetonitrile, 40% methanol, and 20% water with 0.1 N formic acid). To lyse the cell and release intracellular c-di-GMP, resuspension cells were incubated at −20°C for 30 min and centrifuged at 13,000 rpm for 1 min. The supernatant was collected and analyzed by UPLC-MS/MS at Michigan State University. The total amount of c-di-GMP extracted was determined using a standard curve of chemically synthesized c-di-GMP (Enzo Life Sciences, Inc., Farmingdale, NY, USA).

To confirm the UPLC-MS/MS result, a GUS (β-glucuronidase) reporter assay using c-di-GMP responsive riboswitch was performed to detect relative intracellular c-di-GMP concentration (63). *Vibrio cholerae* Vc2 riboswitch cloned into pRS414 were extracted and electroporated into *D. dadantii* wild type and mutants. c-di-GMP levels were evaluated by the β-glucuronidase activity (33). Briefly, overnight bacterial cultures grown in MM were harvested by centrifugation at 10,000 rpm for 3 min. The cell pellet was resuspended in 1× PBS buffer. The cell was treated with 0.1% SDS (sodium dodecyl sulfate) and chloroform in the order. Samples were then centrifuged for 1 min at 10,000 rpm. The upper fraction was collected and mixed with PBS buffer. About 10 µL of 10 mM methylumbelliferyl β-d-glucuronide (MUG) was added, and the mixture was measured at the excitation of 365 nm and emission at 455 nm at different time points. The Bradford assay (Bio-Rad, Hercules, CA, USA) was also done to standardize the GUS value. Briefly, 700 µL of distilled water and 200 µL of Bradford reagent were mixed with 100 µL of upper fraction from the GUS assay, and absorbance at 595 nm was determined. Since c-di-GMP binds to Vc2 riboswitch to suppress GUS expression, the relative GUS activity is inversely proportional to the cellular c-di-GMP concentration, and the relative c-di-GMP level was measured based on WT as a reference point.

### Swimming motility assay

The swimming motility assay was measured as described previously (16). Overnight bacterial cultures (OD_590_ = 1.0) were harvested, and motility was tested by inoculating 10 µL of culture onto the center of MG plates containing 0.2% agar. All plates were then incubated at 28°C and the diameter of the radial growth was measured after 16 h of swimming.

### Pel activity assay

Extracellular Pel activity of cells grown in MM was measured by spectrometry (64). Overnight bacterial cultures in LB broth were transferred 1:100 to MM media supplemented with 0.1% PGA at 28°C for 16 h; bacterial culture was collected at 15,000 rpm for 2 min and 10 µL of the supernatant was added to 990 µL of Pel reaction buffer (0.05% PGA, 0.1 M Tris-HCl [pH 8.5], and 0.1 mM CaCl_2_, pre-warmed to 30°C). Pel activity was monitored at *A*_230_ over 3 min. Pel activity was calculated based on one unit of Pel activity, which is equal to an increase of 1 × 10^−3^ OD_230_ in 1 min.

### Virulence assay

For virulence assays, *D. dadantii* cells were first grown in LB broth at 28°C for 12 h. The cells were harvested and resuspended in PBS to reach 1 × 10^8^ CFU/mL. Russet potato tubers were used for the maceration assay. For each potato slice, 50 µL of bacterial suspensions was added to the center of a 1 cm thick slice. Three replicate slices were used for each strain. Inoculated potato slices were incubated with 100% humidity at 28°C. To evaluate the necrosis of potato tissue, the soft necrotic tissue was scooped out, and the weight was measured for each sample 30 h post-inoculation.

### Construction of site-specific mutations

The full length of *gcpD* and *cdeR* was cloned into the low copy number plasmid pCL1920 by primers gcpD-F-HindIII/gcpD-R-XbaI and cdeR-F-HindIII/cdeR-R-XbaI, respectively (Table S1). To generate a site-specific deletion mutant in the active site of *gcpD* or the HTH regions of *cdeR*, two sets of primers were used to amplify the regions upstream and downstream of the desired mutation or deletion. The amplified fragments were then combined through overlap extension PCR. The resulting substitution or deletion was confirmed via DNA sequencing. The final PCR product was digested and cloned into the pCL1920 vector, followed by electroporation into *D. dadantii* and its derivative strains.

### Statistical analysis

Means and standard deviations of experimental results were performed using SPSS 25 software (IBM, Armonk, NY, USA). Statistical analysis was calculated using Student’s *t* test (Microsoft, Redmond, WA, USA) or one-way ANOVA with *post hoc* Tukey HSD (Honestly Significant Difference) test.

## SUPPLEMENTAL MATERIAL

Fig. S1 spectrum.02655-24-s0001.tif GcpD is not involved in the transcriptional regulation of RpoN. The promoter of rpoN was measured in the parental strain D. dadantii and ΔgcpD.

Fig. S2 spectrum.02655-24-s0002.tif CdeR regulates the T3SS not through RpoN.

Fig. S3 spectrum.02655-24-s0003.tif Deletion of gcpL reduces c-di-GMP levels. Relative c-di-GMP concentrations were measured in wild-type D. dadantii and ∆gcpL.

Fig. S4 spectrum.02655-24-s0004.tif c-di-GMP levels are crucial for CdeR-mediated T3SS regulation.

Fig. S5 spectrum.02655-24-s0005.tif Protein levels of CdeR and its derivatives.

Supplemental material spectrum.02655-24-s0006.docx Legends for all supplemental material.

Table S1 spectrum.02655-24-s0007.docx Primer information.

ASM does not own the copyrights to Supplemental Material that may be linked to, or accessed through, an article. The authors have granted ASM a non-exclusive, world-wide license to publish the Supplemental Material files. Please contact the corresponding author directly for reuse.
