# Supplementary figures and images for: A novel transcriptional regulator, CdeR, modulates the type III secretion system via c-di-GMP signaling in Dickeya dadantii
Source: Microbiol Spectr. 2025 Mar 5;13(4):e02655-24. doi: 10.1128/spectrum.02655-24 (PMC11960120; doi:10.1128/spectrum.02655-24)

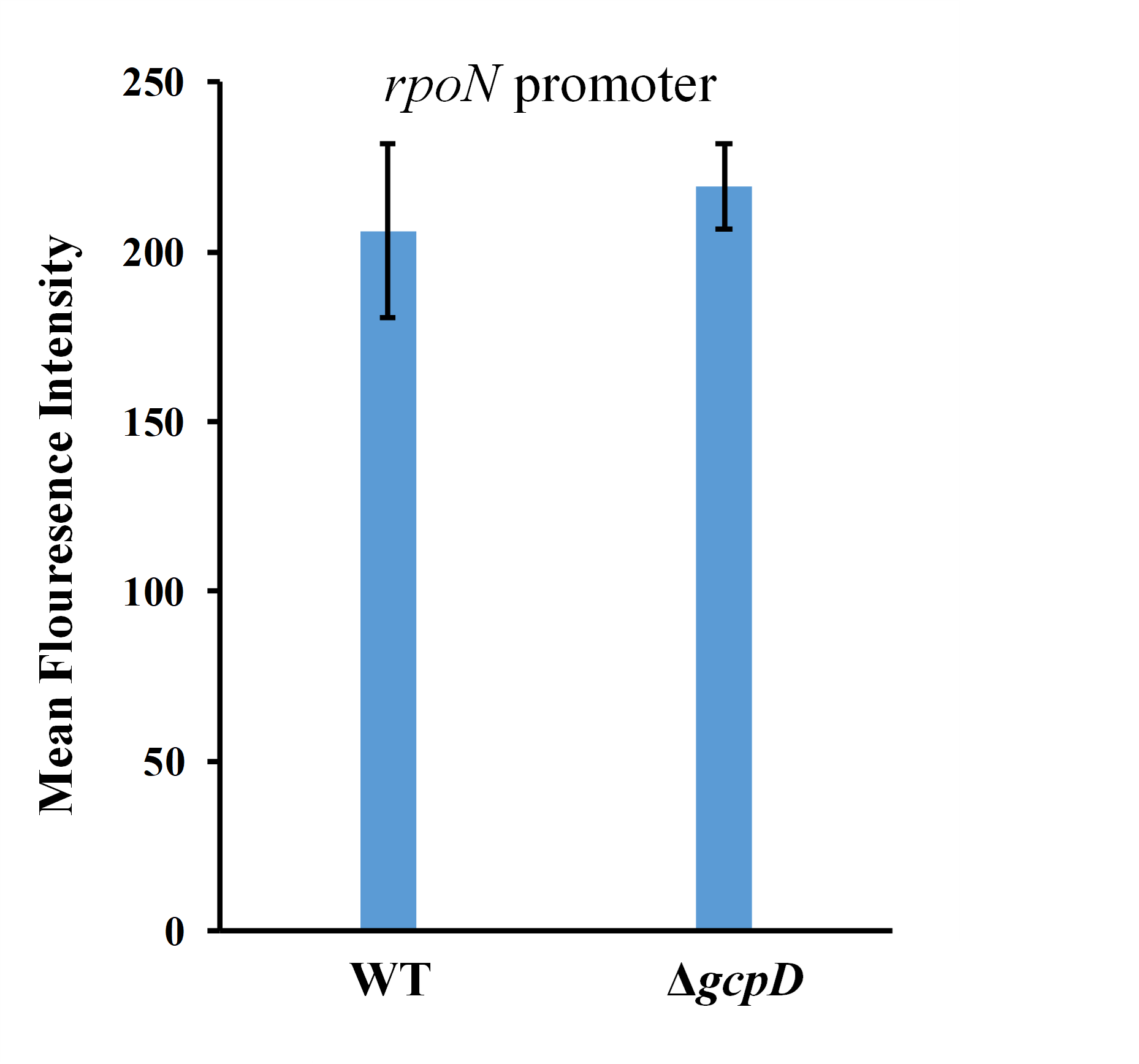

Supplement: Fig. S1 — GcpD is not involved in the transcriptional regulation of RpoN. The promoter of rpoN was measured in the parental strain D. dadantii and ΔgcpD. [file spectrum.02655-24-s0001.tif]

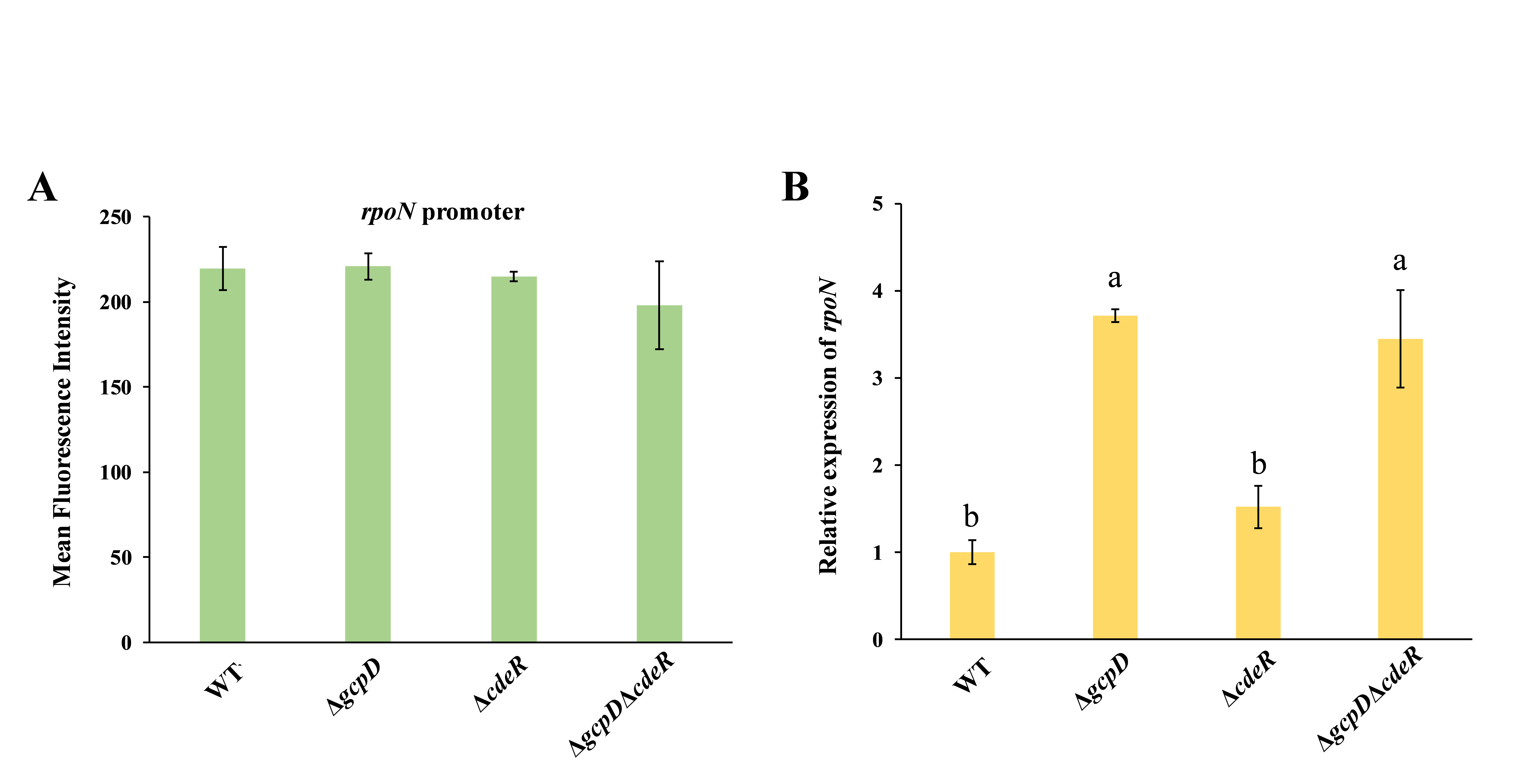

Supplement: Fig. S2 — CdeR regulates the T3SS not through RpoN. [file spectrum.02655-24-s0002.tif]

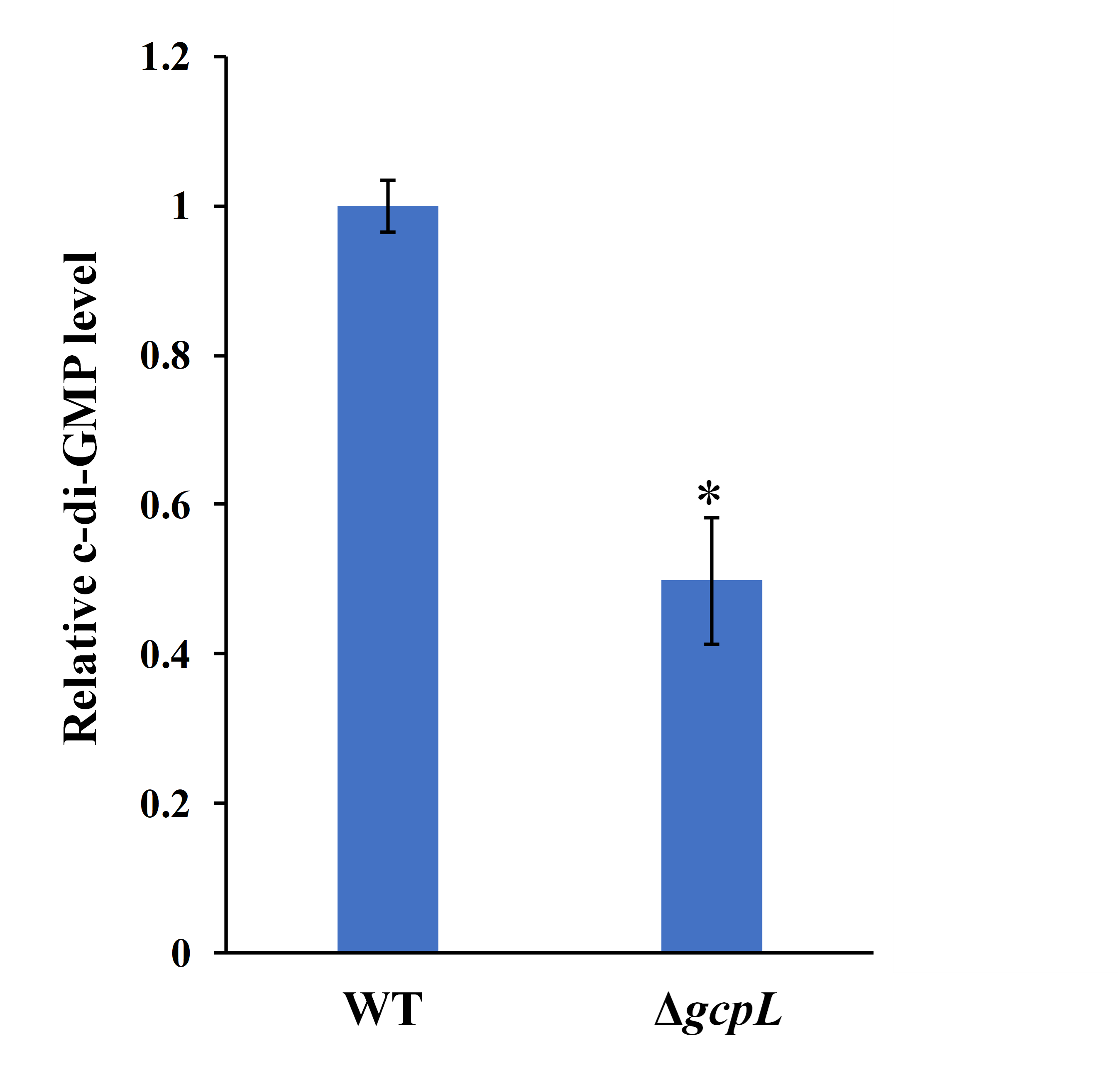

Supplement: Fig. S3 — Deletion of gcpL reduces c-di-GMP levels. Relative c-di-GMP concentrations were measured in wild-type D. dadantii and ∆gcpL. [file spectrum.02655-24-s0003.tif]

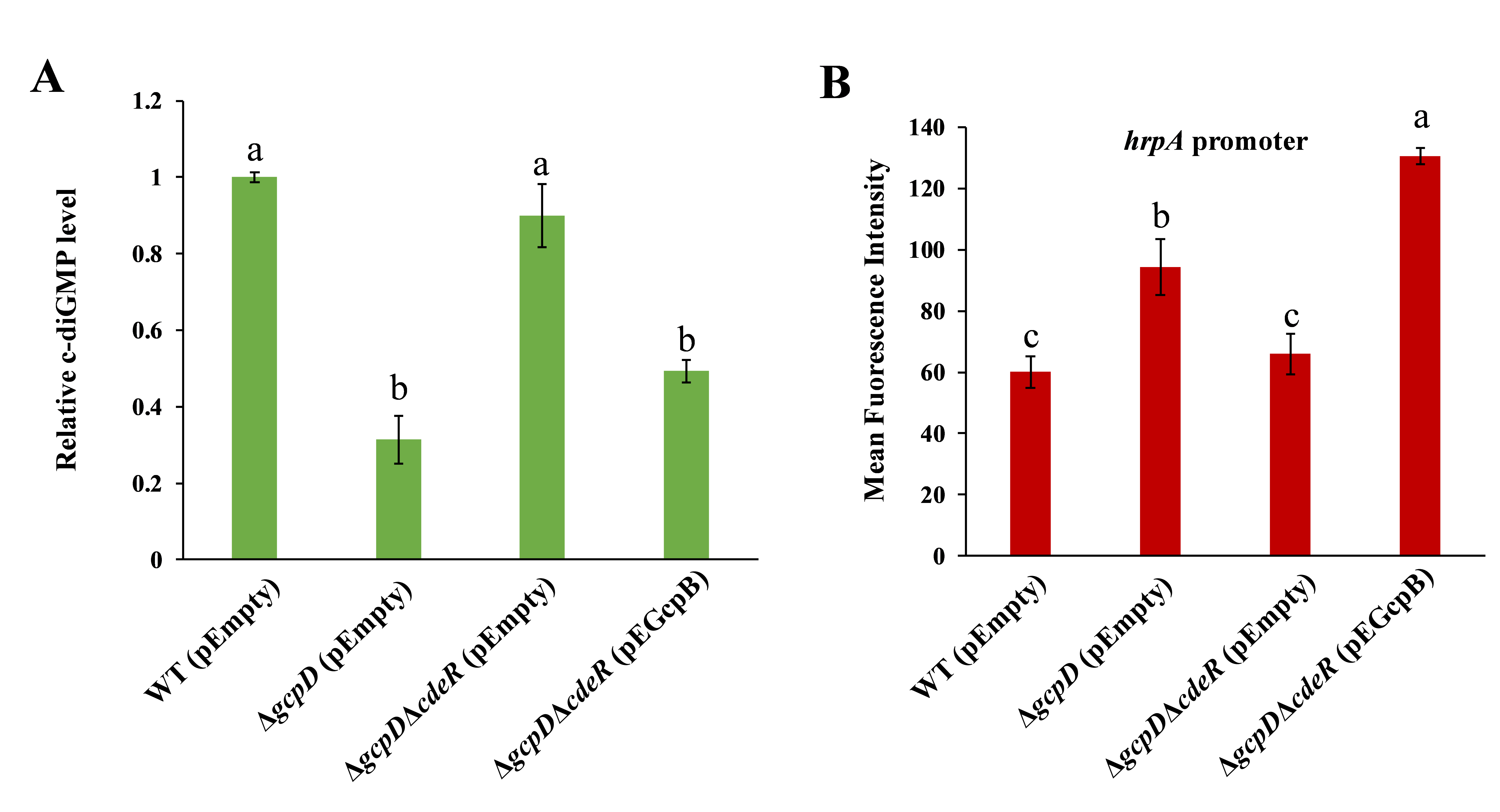

Supplement: Fig. S4 — c-di-GMP levels are crucial for CdeR-mediated T3SS regulation. [file spectrum.02655-24-s0004.tif]

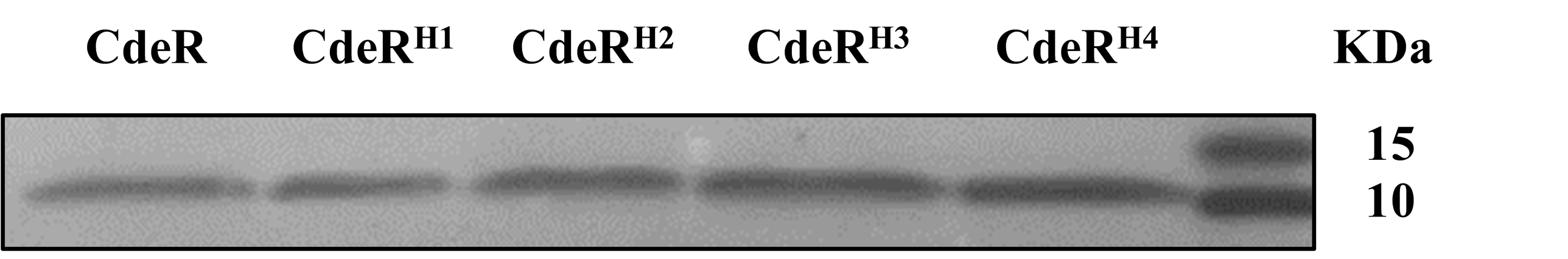

Supplement: Fig. S5 — Protein levels of CdeR and its derivatives. [file spectrum.02655-24-s0005.tif]
