# Supplementary material for: A novel transcriptional regulator, CdeR, modulates the type III secretion system via c-di-GMP signaling in Dickeya dadantii
Source: Microbiol Spectr. 2025 Mar 5;13(4):e02655-24. doi: 10.1128/spectrum.02655-24 (PMC11960120; doi:10.1128/spectrum.02655-24)
Supplement: Supplemental material — Legends for all supplemental material. [file spectrum.02655-24-s0006.docx]

**Supplementary information**

**Table S1. Primer information.**

**Figure S1. GcpD is not involved in the transcriptional regulation of RpoN.** The promoter of *rpoN* was measured in the parental strain *D. dadantii* and Δ*gcpD*. Values are representative of three experiments, and three replicates were used for each experiment. Error bars indicate standard errors of the means.

**Figure S2. CdeR regulates the T3SS not through RpoN.** (A) The promoter activities and (B) RNA levels of *rpoN* were measured in wild-type *D. dadantii*, Δ*gcpD*, Δ*cdeR*, and Δ*gcpD*Δ*cdeR.* Similar results were observed in three independent experiments. The lowercase letters indicate statistically significant differences between treatments (*P* < 0.05) by one-way ANOVA.

**Figure S3. Deletion of *gcpL* reduces c-di-GMP levels.** Relative c-di-GMP concentrations were measured in wild-type *D. dadantii* and ∆*gcpL*. Similar results were observed in three independent experiments. Each experiment includes three replicates. Error bars indicate standard errors of the means. Asterisks indicate statistically significant differences in the means (*P* < 0.05 by Student's t-test).

**Figure S4. c-di-GMP levels are crucial for CdeR-mediated T3SS regulation.** (A) Relative c-di-GMP concentrations and (B) *hrpA* promoter activities were measured in wild-type *D. dadantii* harboring the empty vector pCL1920, Δ*gcpD* harboring pCL1920, Δ*gcpD*Δ*cdeR* harboring pCL1920, and ΔgcpDΔcdeR harboring pCL1920-*egcpB.* The lowercase letters represent different treatment groups with significant statistical differences, whereas treatments with no significant differences were shown the same letters (*P* < 0.05) by one-way ANOVA.

**Figure S5. Protein levels of CdeR and its derivatives.** SDS PAGE analysis of wild-type CdeR, CdeR^H1^, CdeR^H2^, CdeR^H3^, and CdeR^H4^.
