# Supplementary material for: A novel transcriptional regulator, CdeR, modulates the type III secretion system via c-di-GMP signaling in Dickeya dadantii
Source: Microbiol Spectr. 2025 Mar 5;13(4):e02655-24. doi: 10.1128/spectrum.02655-24 (PMC11960120; doi:10.1128/spectrum.02655-24)
Supplement: Table S1 — Primer information. [file spectrum.02655-24-s0007.docx]

| **Primers** | **Sequences (5’-3’)** | **use** |
| --- | --- | --- |
| *cdeR-*A-XhoI  *cdeR-*B  *cdeR*-C  *cdeR*-D-NotI | AATACTCGAGTGAGTTAGATTGGCTTGTA  GAAGCAGCTCCAGCCTACACCGTGCCAGTCTTTATCAATCA  CTAAGGAGGATATTCATATGTTCGCCTGCGGAAATCTG  AATATTATGCGGCCGCTTCAGAAGCCTTACCAACG | *cdeR* deletion |
| *gcpD-*A*-*XhoI  *gcpD*-B  *gcpD*-C  *gcpD*-NotI | AATACTCGAGTGCTCCAACCTGTTTCTGGG  GAAGCAGCTCCAGCCTACACAAGATATTCAGCATGCGCGC  CTAAGGAGGATATTCATATGAACGCCAACGAGTTGATCCG  AATATTATGCGGCCGCTCGAAGGATTGCTGGAATGC | *gcpD* deletion |
| *gcpD*-D525A-1  *gcpD*-D525A-2 | CCGTCTGGCAGGCGCCGAATTCCTGATG  CCATCAGGAATTCGGCGCCTGCCAGACGG | site-directed *gcpD* |
| *gcpD*-for- HindIII  *gcpD*-rev-XbaI | AAAAAAAGCTTAATCATCACCAGGTACCATAAGGTTTC  AAAAATCTAGATCAGGATGGGGAGGTATGACGACG | *gcpD* complementation |
| *cdeR-*for*-* HindIII  *cdeR-*rev*-*XbaI | AAAAAAAGCTTAATGTGAGGCTACAGAATAAGGATCGTG AAAAATCTAGATTAACGATTTTCTGACTTGCGGTAACG | *cdeR* complementation |
| *gcpL*-F  *gcpL*-R | AAACGTTCTCGATACCCATCGTGCG GCGGGTTATCGTACTGACGTTAACC | *gcpL*  qPCR |
| *hrpA*-F  *hrpA*-R | ATGATGGGACTTTCTAACGCAGCAG  TCAGAACTGAATAGCTTTGGCCGCT | *hrpA*  qPCR |
| *rplU*-F  *rplU*-R | CAAAGTGGTGGTAAACAACACCGAG  TTTTAACTTTCTCGCCACGACCGTG | *rplU*  qPCR |
| *slyA*-F  *slyA*-R | ACGCACTGGGTCACACTGCATAATA  ACAGCCTGAATGATAGGATCCGCCA | *slyA*  qPCR |
| PADIIAGLRKR*-*F  PADIIAGLRKR*-*R | GGCGATGATTGATAAAGACTGGCACGGAACAACACTGGCAGCGGTTTCGC  GCGAAACCGCTGCCAGTGTTGTTCCGTGCCAGTCTTTATCAATCATCGCC | *cdeR*^H1^ |
| LAAVSRNA*-*-F  LAAVSRNA-R | CGGGTTAAGAAAACGAGGAACAACAGGGTTAGCGTCTTCCACGCTGGCAA  TTGCCAGCGTGGAAGACGCTAACCCTGTTGTTCCTCGTTTTCTTAACCCG | *cdeR*^H2^ |
| ASSTLANR-F  ASSTLANR*-*R | AGCGGTTTCGCGTAACGCGGGGTTAGCGCTAACAAGACACTGGCCTAAAG  CTTTAGGCCAGTGTCTTGTTAGCGCTAACCCCGCGTTACGCGAAACCGCT | *cdeR*^H3^ |
| PKGEKLIAEAL-F  PKGEKLIAEAL-R | GGCAAATGCGCTAACAAGACACTGGGGTGTTTCGCCTGCGGAAATCTGGC  GCCAGATTTCCGCAGGCGAAACACCCCAGTGTCTTGTTAGCGCATTTGCC | *cdeR*^H4^ |
| P1  P2 | GCGATTGTGTAGGCTGGAGCTGCTTC  GCTGACATGGGAATTAGCCATGGTCC | Kanamycin cassette amplification from pKD4 plasmid |
|  |  |  |
